# Eustachian Tube Dysfunction Improvement Secondary to Moderate Weight Loss: Case Report

**DOI:** 10.7759/cureus.24554

**Published:** 2022-04-28

**Authors:** Mohammad H Shaheen, Sara Bayounos, Elsaeid M Thabet, Bassam Al-zuraiqi, Khalid Badr, Saeed A Alghamdi, Fares E AlGhamdi

**Affiliations:** 1 Otolaryngology - Head and Neck Surgery, King Abdullah Medical City, Makkah, SAU; 2 Otolaryngology - Head and Neck Surgery, Al-Noor Specialist Hospital, Makkah, SAU

**Keywords:** secondary to moderate weight loss, eustachian tube function, eustachian tube dysfunction improvement, moderate weight loss, eustachian tube dysfunction

## Abstract

The Eustachian tube (ET) is an osteocartilaginous canal that connects the middle ear to the nasopharynx. It is one of the critical structures essential for middle ear functions. ET dysfunction causes discomfort in the affected ear and decreased hearing. This is the first case report of improving hearing and middle ear aeration and ET function secondary to body weight reduction. A 27-year-old male patient presented to the otology clinic complaining of decreased hearing for two years. Initial ear examination revealed retraction of TM on the left ear with two retraction pockets, and on the right ear, the TM was dull with one retraction pocket. The patient reported losing some of his body weight during those six months. Ear examination revealed improvement in the TM retraction in both ears. The improvement in hearing was evidenced by a serial audiogram, while the middle ear aeration was evidenced by clinical examination.

## Introduction

The Eustachian tube (ET) is an osteocartilaginous canal that connects the middle ear to the nasopharynx. It is one of the critical structures essential for middle ear functions. It is responsible for the processes of protection, aeration, and draining [1] Autophony, tinnitus, and the sensation of aural fullness are all symptoms of tubal dysfunction [1]. Eustachian tube dysfunction (ETD) can be caused by various factors. Infectious, allergic, and mechanical (obstructive) reactions, exposure to an irritant, laryngeal reflux, congenital malformation, and iatrogenic are a few causes. Some of the more common etiologies have also been investigated; however, this is not an exhaustive list.

It has been found that a healthy and functional ET has been associated with a reduced incidence of ear complications secondary to ETD [2]. Patients who had undergone endoscopic sinuous surgery (ESS) got relief from their otologic symptoms related to ETD [3]. Allergic rhinitis and viral infections are well-known causes of ETD, which, in turn, can lead to otitis media effusion [4]. Adenoid enlargement has been historically related to ETD. Recently, it has been found that it hindered mucociliary clearance from the tube through the metaplastic non-ciliated epithelium and connective tissue fibrosis associated with surrounding adenoid tissue [5]. The ciliary beat frequency of the ET mucosa was shown to be lower in smokers compared to nonsmokers [6]. External beam radiation therapy for nasopharyngeal cancer has negative effects on structures surrounding the tumor, particularly the ET. Patients with early and late middle ear diseases as a result of iatrogenic ET damage such as after adenoidectomy have been reported [7].

ETD causes discomfort in the affected ear and decreased hearing. Effusion, atelectasis, and retraction with cholesteatoma are well-known sequelae of ETD. Also, patients with ETD are at a high risk of tympanic membrane (TM) perforation secondary to atmospheric pressure change (Barotrauma) [8].

This is the first case report of an improvement in hearing and middle ear aeration and ET function secondary to body weight reduction. The improvement in hearing was evidenced by a serial audiogram, while the middle ear aeration was evidenced by clinical examination.

## Case presentation

**Figure 1:**
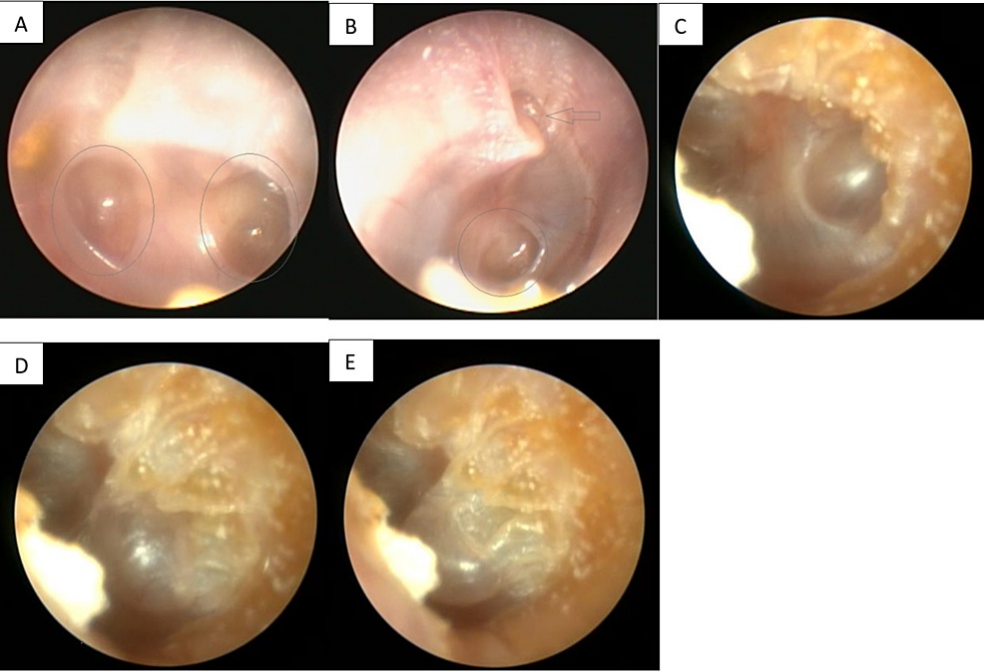
(A) Left ear on presentation, with retraction pockets indicated by circles. (B) Right ear after weight loss. (C) Left ear after weight loss, with the retraction pocket inflated by Valsalva (was not achievable before weight loss). (D and E) Left ear after weight loss with the retraction pocket inflated by Valsalva.

**Figure 2:**
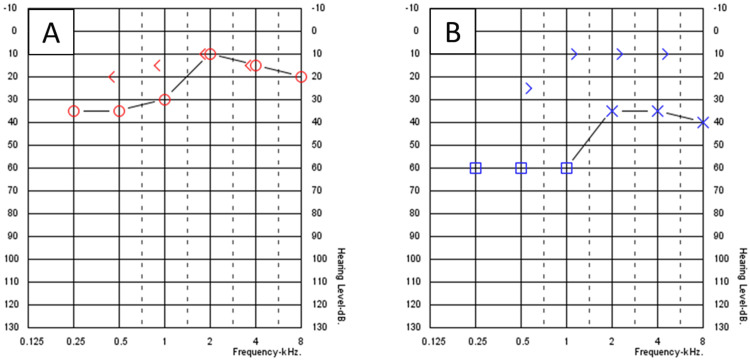
Pure tone audiometry after insertion the right ventilation tube. (A) Right ear. (B) Left ear.

**Figure 3:**
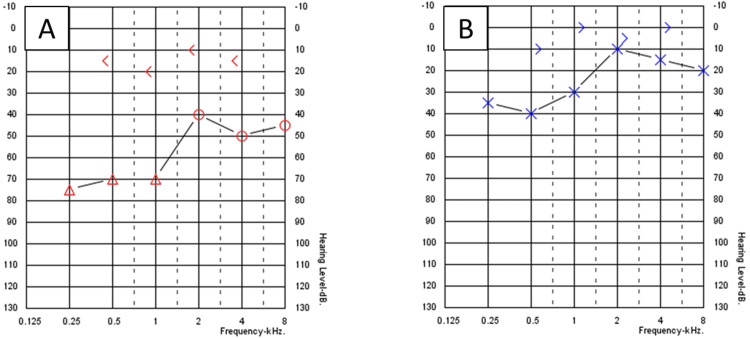
Pure tone audiometry after extrusion of the right ventilation tube. (A) Right ear. (B) Left ear.

**Figure 4:**
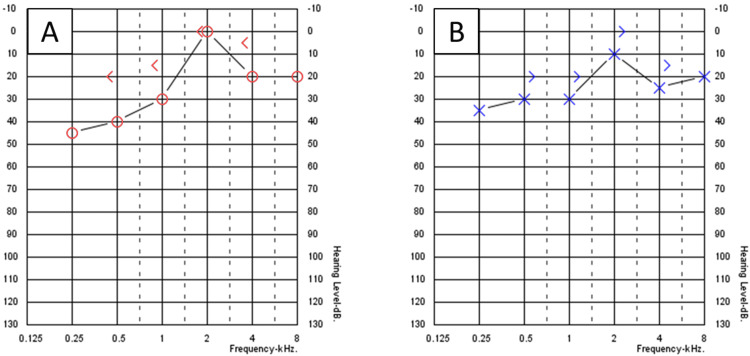
Pure tone audiometry after weight loss. (A) Right ear. (B) Left ear.

A 27-year-old male patient not known to have any medical illness apart from allergic rhinitis presented to the otology clinic complaining of decreased hearing for two years. There was no ear pain, ear discharge, autophony, tinnitus, or aural fullness sensation. The patient revealed a history of pressure equalization tube (PET) insertion in both ears 12 years prior to this presentation. Initial ear examination revealed retraction of TM on the left ear with two retraction pockets (Figure 1A), and on the right ear, the TM was dull with one retraction pocket (Figure 1B). There was no auricular or external auditory canal deformity in both ears. Nasal examination showed bilateral congested hypertrophied inferior turbinate with thin clear secretion. Examination of the nasopharynx revealed edematous mucosa, but no discrete mass could be seen. His body weight was 124 kg and height was 170 cm. Pure tone audiometry (PTA) showed bilateral moderate conductive hearing loss (CHL), with an excellent speech discrimination in both ears. Tympanometry was type B at the right ear and type A at the left ear; intranasal corticosteroid spray and normal saline spray were prescribed. After one week from the presentation, myringotomy and PET insertion in the right ear only were performed to obtain temporary relief of the condition as the patient preferred not to undergo any major procedure at that time. The PTA post-tube insertion showed closure of the air-bone gap (ABG) in the right ear, while the left ear audiometry was still the same (Figure 2). The PET was extruded from the right ear seven months post-operation with complete healing of TM, and PTA revealed moderate-to-severe CHL in the right ear and mild CHL in the left ear (Figure 3). The patient was offered surgical intervention (cartilage tympanoplasty), but he refused and preferred using hearing aids. Six months later, there was significant improvement in hearing that made the patient stop using his hearing aids. Also, the patient reported losing some of his body weight during those six months; it reduced by 10 kg and his weight became 114 kg. Ear examination revealed improvement in the TM retraction in both ears, but one retraction pocket on the left ear was inflated (reversible) by Valsalva (Figures 1C-1E). A follow-up audiogram revealed closure of the ABG in both ears (Figure 4).

## Discussion

There are three physiological functions for the ET: (1) regulation of the middle ear pressure, (2) safeguarding of the middle ear from nasopharyngeal pathogens, and (3) drainage of the middle ear. The ET length has been reported to be between 31 mm and 38 mm [9]. The normal alignment of the ET is anterior, downward, and with a medial rotation in adults, while in children, it is shorter, narrower, and more horizontal. With this location, the ET establishes an angle of about 30-40 degrees and 45 degrees with the horizontal and sagittal planes, respectively [10]. Usually, the ET stays closed and only opens when required to balance pressure. Other functions include drainage of middle ear space fluid while at the same time preventing nasopharyngeal secretions from refluxing into the middle ear space. The physiological function of the ET was studied by Ghadiali et al. [11] who discovered that the ET opening was extremely sensitive to applied muscular stresses but rather insensitive to cartilage elastic characteristics. Luminal dilation of the ET was caused by muscle contraction causing medial-superior rotation of the medial lamina, which, in turn, caused deformation of fatty tissue surrounding the ET (Ostmann's fat pad), according to their analysis of muscle forces (tensor and levator veli palatini) and soft tissue elastic properties [11]. Functional failure of the ET resulted in increased negative middle ear pressure, TM atelectasis, creation of a retraction pocket in the attic or posterior-superior quadrant, and otitis media effusion [12].

Weight loss is linked to lower blood pressure and glucose levels, as well as a lower incidence of hypertension and non-insulin-dependent diabetic mellitus, according to observational studies [13] and interventional studies [14]. The benefits of moderate weight loss on the cardiovascular risk factors associated with obesity are now well documented [15]. Fasting glycemia, glycosylated hemoglobin (HBA1c), systolic and diastolic blood pressure, and plasma lipid profile all improve with a 5-10% weight loss [15]. The benefits of a moderate weight loss on hypertension and blood lipids have also been clearly studied. According to MacMahon et al.'s meta-analysis, each kilogram of weight loss reduced systolic and diastolic blood pressure by 0.68 and 0.34mmHg, respectively [16]. Dattilo and Kris-Etherton's meta-analysis found that losing 1 kg lowers blood cholesterol by 2.28 mg/dL, low-density lipoprotein (LDL) cholesterol by 0.91 mg/dL, and triglycerides by 1.54 mg/dL [17]. These favorable effects appear to apply not only to absolute plasma lipid levels but also to several essential qualitative aspects of lipoproteins, according to new studies. According to Vasankari et al.’s study, a one-year weight loss intervention program was linked to a decrease in the plasma concentration of oxidized-LDL particles [18]. The quantity of weight lost was proportional to the lowering of the most atherogenic of LDL particles. A reduction of 33% in the ratio of oxidized LDL to total LDL was associated with a weight loss of roughly 10% of the initial body weight.

Patulous ET is defined as a patency abnormality that causes autophony. The patulous ET symptoms were first reported in 1858 by Jago [19]. Patients commonly describe their affected ear as being blocked, in confusion of the patulous ET and tubal blockage or dysfunction. It can occur spontaneously or be triggered by physical activity, prolonged talking, or the use of nasal or oral decongestants. The majority of patients report that their autophony is intermittent, although in severe situations, the symptoms might last for hours. Placing the head in a dependent position or lying supine for some time can often relieve autophony, at least briefly. Sniffing inward against a closed nostril to induce negative middle ear pressure, ipsilateral internal jugular vein compression, and the presence of an upper respiratory tract illness or allergies have all been documented to provide temporary relief. The loss of tissue within the cartilaginous region of ET is assumed to be the cause of patulous ET. According to Pascoto et al.’s study [20], rapid weight loss, such as that seen after bariatric surgery, can cause symptoms of ETD (patulous ET), most likely due to the loss of peritubular fat.

This report shows that the patient's body mass index (BMI) was 42.9, which is classified as obesity class 3 depending on the WHO classification for obesity, and his BMI reduced to 39.4 changing his obesity class to class 2. This moderate weight loss of roughly 8% of the initial body weight has a favorable effect on ETD as well as on other well-known systemic conditions such as hyperlipidemia and diabetes. This observation can be the basis for other studies that may prove an association or a cause-and-effect relationship. This will be novel conservative management for ETD without any serious side effects on the patient.

## Conclusions

This report shows that the moderate weight loss of roughly 8% of the initial body weight has a favorable effect on ETD as well as on other well-known systemic conditions such as hyperlipidemia and diabetes. This observation can be the basis for other studies that may prove an association or a cause-and-effect relationship. This will be novel conservative management for ETD without any serious side effects on the patient.
